# Perioperative risk factors for new-onset postoperative atrial fibrillation after coronary artery bypass grafting: a systematic review

**DOI:** 10.1186/s12872-021-02224-x

**Published:** 2021-09-03

**Authors:** Eun Ji Seo, Joonhwa Hong, Hyeon-Ju Lee, Youn-Jung Son

**Affiliations:** 1 Ajou University College of Nursing and Research Institute of Nursing Science, Suwon, 16499 Republic of Korea; 2 Department of Thoracic and Cardiovascular Surgery, Chung-Ang University, Seoul, 06974 Republic of Korea; 3 Department of Nursing, Tongmyoung University, Busan, 48520 Republic of Korea; 4 Red Cross College of Nursing, Chung-Ang University, 84 Heukseok-ro Dongjak-Gu, Seoul, 06974 Republic of Korea

**Keywords:** Predictor, Atrial fibrillation, Coronary artery bypass graft, Systematic review

## Abstract

**Background:**

Postoperative atrial fibrillation (POAF) is the most common cardiac dysrhythmia to occur after coronary artery bypass grafting (CABG). However, the risk factors for new-onset POAF after CABG during the perioperative period have yet to be clearly defined. Accordingly, the aim of our systematic review was to evaluate the perioperative predictors of new-onset POAF after isolated CABG.

**Method:**

Our review methods adhered to the Preferred Reporting Items for Systematic Reviews and Meta-Analyses guideline. We searched seven electronic databases (PubMed, Embase, CINAHL, PsycArticles, Cochrane, Web of Science, and SCOPUS) to identify all relevant English articles published up to January 2020. Identified studies were screened independently by two researchers for selection, according to predefined criteria. The Newcastle–Ottawa Scale was used to evaluate the quality of studies retained.

**Results:**

After screening, nine studies were retained for analysis, including 4798 patients, of whom 1555 (32.4%) experienced new-onset POAF after CABG. The incidence rate of new-onset POAF ranged between 17.3% and 47.4%. The following risk factors were identified: old age (*p* < 0.001), a high preoperative serum creatinine level (*p* = 0.001), a low preoperative hemoglobin level (*p* = 0.007), a low left ventricle ejection fraction in Asian patients (*p* = 0.001), essential hypertension (*p* < 0.001), chronic obstructive pulmonary disease (*p* = 0.010), renal failure (*p* = 0.009), cardiopulmonary bypass use (*p* = 0.002), perfusion time (*p* = 0.017), postoperative use of inotropes (*p* < 0.001), postoperative renal failure (*p* = 0.001), and re-operation (*p* = 0.005). All studies included in the analysis were of good quality.

**Conclusions:**

The risk factors identified in our review could be used to improve monitoring of at-risk patients for early detection and treatment of new-onset POAF after CABG, reducing the risk of other complications and negative clinical outcomes.

**Supplementary Information:**

The online version contains supplementary material available at 10.1186/s12872-021-02224-x.

## Background

Coronary artery bypass grafting (CABG) is the standard of care for the treatment of advanced coronary artery disease [1, 2]. Despite its value, CABG is associated with a high risk of postoperative cardiac and non-cardiac complications, including dysrhythmia, the need for re-operation, cognitive decline, and mortality [3–5]. New-onset postoperative atrial fibrillation (POAF) is the most common cardiac dysrhythmia to occur after CABG [6, 7]. Defined as POAF developing within two to four days after CABG, new-onset POAF is identified in 10–40% of patients in the early postoperative period after CABG, with the peak onset at two days postoperatively [4, 8]. POAF after CABG increases the length of postoperative hospital stay and is associated with an increased risk of hospital readmission, stroke, and early and late mortality [8–10]. Recent research has also indicated that new-onset POAF after CABG has a long-term thromboembolic risk profile similar to that of non-valvular atrial fibrillation (AF) [11, 12]. Accordingly, identification of patients who are at risk of new-onset POAF after CABG is clinically important to ensure adequate precautions during the perioperative period to optimize clinical outcomes.

The following predisposing factors for POAF after CABG have previously been identified: advanced age, obesity, and comorbidities, such as hypertension, diabetes mellitus, and chronic obstructive pulmonary disease (COPD) [4, 7, 13, 14]. However, the risk factors for new-onset POAF after CABG remain inconclusive. Systematic reviews regarding the relationship between POAF and adverse outcomes after CABG have largely focused on mortality [8, 12, 15]. One systematic review which did seek to identify risk factors for POAF after CABG included studies for only on-pump CABG with all types of cohort study designs [4]. The impact of cardiopulmonary bypass (CPB) on the clinical outcomes of CABG, including POAF, is still being debated [16]. As risk models based only on preoperative risk factors cannot identify all patients who develop POAF [17], it is necessary to identify the risk factors that can be continuously monitored during and after CABG for optimal care.

Therefore, we aimed to identify the pre-, peri-, and postoperative predictors of new-onset POAF among patients who underwent isolated CABG through a systematic review of research evidence. To control for confounding variables on the possible causative pathway between identified factors and new-onset POAF, only prospective studies were included in our review and meta-analysis [18].

## Methods

Our systematic review was performed in accordance with the Preferred Reporting Items for Systematic Reviews and Meta-Analysis and Meta-Analysis of Observational Studies in Epidemiology guidelines [19, 20]. The following question, developed using the Patient, Interest, Comparison, Outcomes, and Study Design (PICOS) format, guided our systematic review: What are the risk factors (Interest) for new-onset POAF (Outcomes) after CABG (Patients) identified in a prospective study (Study Design)? Note that as clinical trials were not selected in our systematic review, the “comparison” term was not included.

### Search strategy

As a first step, we searched the Cochrane Library and Joanna Briggs Institute EBP databases, as well as the International Prospective Register of Systematic Reviews, to identify existing or ongoing reviews on our specific topic. Subsequently, we performed a systematic search of the following electronic databases to identify relevant evidence, up to January 2020: PubMed, Embase, CINAHL, PsycArticles, Cochrane, Web of Science, and SCOPUS. Search terms were developed with the assistance of a medical librarian and individualized for each database. The following Medical Subject Headings or Emtree terms were used: (“CABG” OR “Coronary artery bypass grafting” OR “Coronary artery bypass graft” OR “Thoracic Surgery” OR “Cardiac Surgery” OR “Heart Surgery” OR “CABG surgery” OR “Coronary artery bypass graft surgery” OR “Off pump CABG”) AND (“AF” OR “Atrial Fibrillation”) AND (“Risk factors” OR “risk”). In addition, the reference lists of identified studies were also screened to identify further relevant studies for inclusion. Further, two authors independently performed manual searches to identify studies that could have been missed in the database search.

### Study selection

The inclusion criteria for individual studies were as follows: (1) full text in English, (2) patients ≥ 18 years of age, (3) CABG performed to treat coronary artery disease, and (4) use of a prospective design to explore the risk factors for new-onset POAF after CABG. The exclusion criteria were as follows: (1) study protocols, reviews, commentaries, editorials, and letters to the editor, (2) patients with a history of AF before CABG, (3) patients who underwent other heart surgery in addition to CABG, (4) CABG for congenital heart diseases, (5) identified effect of preexisting AF or POAF on postoperative outcomes, (6) unable to extract unadjusted data for risk factors according to POAF occurrence, (7) focus on a specific subgroup of the population of interest, and (8) evaluation of the effectiveness of drugs or herbs as treatment.

**Fig. 1:**
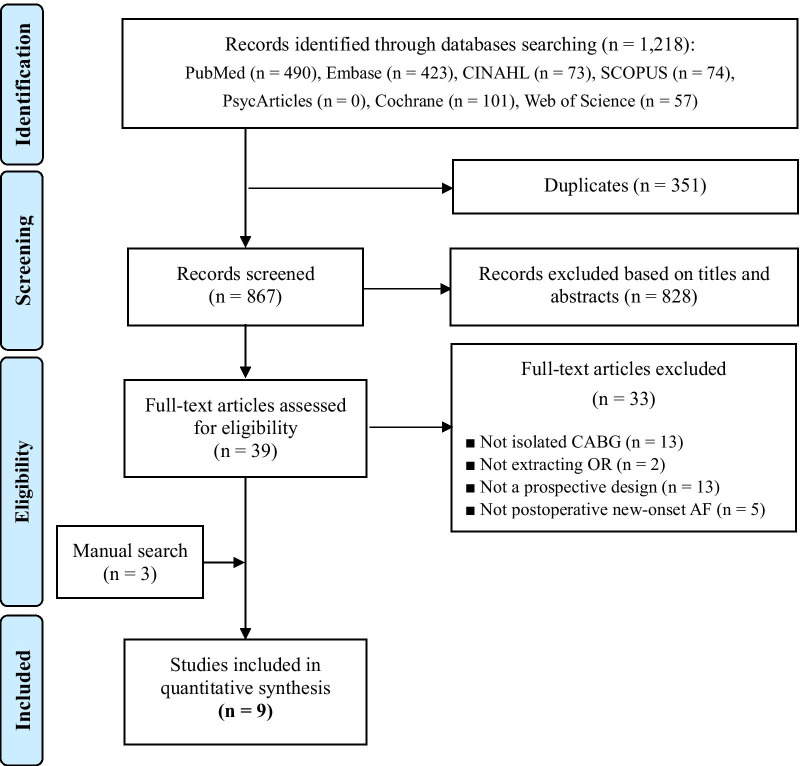
The flow diagram of the study selection process

After excluding duplicates, the title and abstract of 867 articles were reviewed independently by two authors and conflicts were resolved through discussion. Of these, 828 articles were excluded, and a full-text review was completed for the remaining 39 articles. After full-text review, 33 articles were further excluded. Our manual search of the reference lists identified an additional three articles [21–29]. The flow diagram for the selection of the nine studies included in our analysis is shown in Fig. 1.

### Assessment of risk of bias

The Newcastle–Ottawa Scale (NOS) [30] was used to evaluate the quality of selected studies. The NOS is an 8-item scale, assessing the following three domains: selection, comparability, and outcome. The maximum scale score of 9 is subdivided as follows: 4 points for selection, 2 for comparability, and 3 for outcome. Interpretation of the total score is as follows: 7–9, good quality; 4–6, fair quality; and 0–3, poor quality. The NOS was scored independently by two authors and conflicts were resolved through discussion.

### Data extraction

**Fig. 2:**
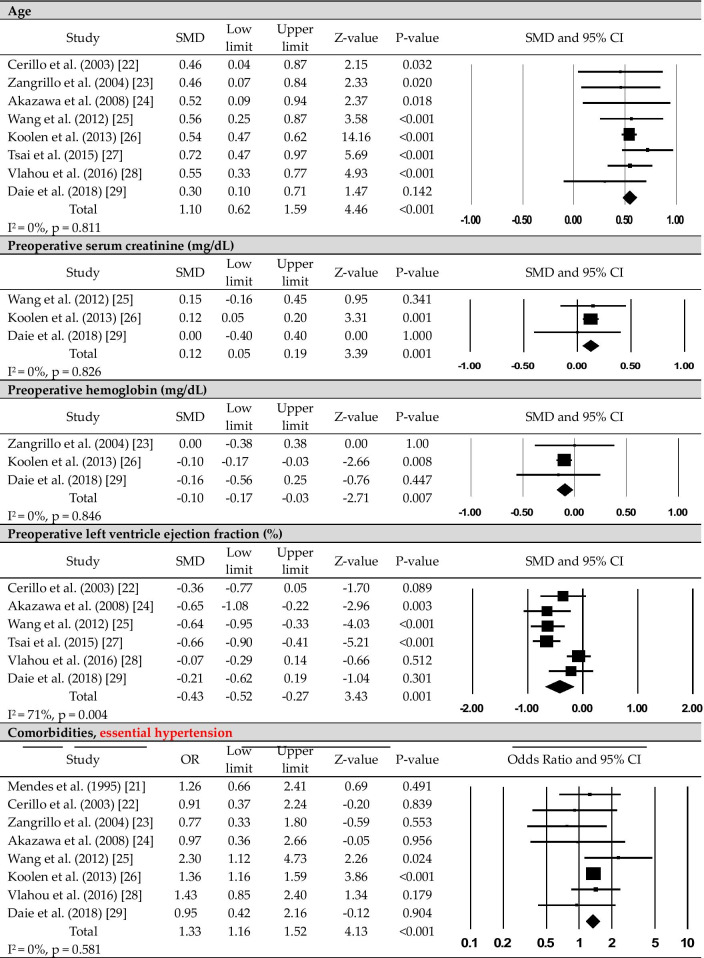

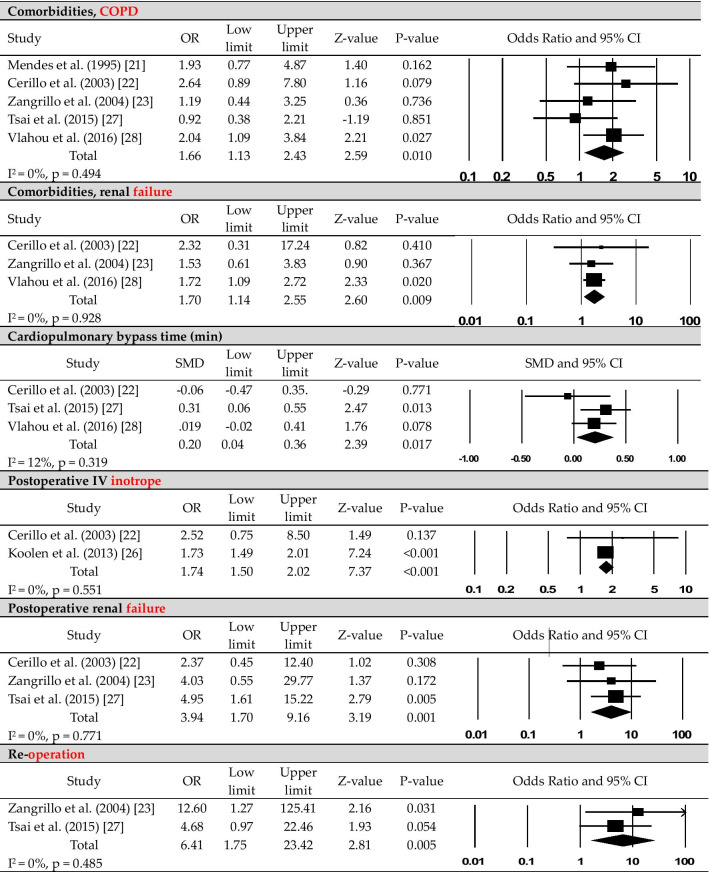
The forest plots of the significant risk factors for new-onset postoperative atrial fibrillation after coronary artery bypass grafting

The following information was reviewed independently by two authors and extracted as characteristics and main results from the nine studies included in the analysis (Table 1): name of first author, year of publication, study country, median time to new-onset POAF, and participant characteristics (e.g., sample size, mean age, sex). To determine the effect size of the factors associated with new-onset POAF after CABG, a meta-analysis was performed; the synthesis of these outcomes is summarized in Table 2 and Fig. 2.

**Table 1:** Characteristics of included studies (N = 9)

Authors (publication year)/country	Sample size	Type of CABG	Time to onset of POAF	Sample characteristics	
				New-onset POAF	Sinus rhythm
Mendes et al. [21]/USA	168	On-pump	3.4 ± 0.2 days after surgery(mean)	N = 57 (33.9%) Mean age: 69.7 years M: 72.0% F: 28.0%	N = 111 (66.1%) Mean age: 62.1 years M: 65.0% F: 35.0%
Cerillo et al. [22]/Italy	107	Both	3 days after surgery (median)	N = 33 (30.8%) Mean age: 70.2 years M: 78.8% F: 21.2%	N = 74 (69.2%) Mean age: 65.8 years M: 68.9% F: 31.1%
Zangrillo et al. [23]/Italy	160	Off-pump	Unreported	N = 33 (20.6%) Mean age: 68.0 years M: 87.9% F: 12.1%	N = 127 (79.4%) Mean age: 64.0 years M: 84.3% F: 15.7%
Akazawa et al. [24]/Japan	150	Off-pump	48 h after surgery (mean)	N = 26 (17.3%) Mean age: 71.0 years M: 88.0% F: 12.0%	N = 124 (82.7%) Mean age: 66.0 years M: 79.0% F: 21.0%
Wang et al. [25]/China	197	Unreported	4 days after surgery	N = 60 (30.5%) Mean age: 71.0 years M: 68.3% F: 31.7%	N = 137 (69.5%) Mean age: 64.0 years M: 69.3% F: 30.7%
Koolen et al. [26]/Netherlands	3148	Both	Unreported	N = 1080 (34.3%) Mean age: 69.8 years M: 77.0% F: 23.0%	N = 2068 (65.7%) Mean age: 64.7 years M: 79.0% F: 21.0%
Tsai et al. [27]/Taiwan	266	Both	Unreported	N = 126 (47.4%) Mean age: 69.9 years M: 71.4% F: 28.6%	N = 140 (52.6%) Mean age: 61.8 years M: 82.1% F: 17.9%
Vlahou et al. [28]/Greece	446	On-pump	Unreported	N = 111 (24.9%) Mean age: 68.1 years M: 83.8% F: 16.2%	N = 335 (75.1%) Mean age: 63.5 years M: 85.9% F: 14.1%
Daie et al. [29]/Iran	156	Unreported	Unreported	N = 29 (18.6%) Mean age: 63.9 years M: 62.1% F: 37.9%	N = 127 (81.4%) Mean age: 61.2 years M: 68.5% F: 31.5%

CABG, coronary artery bypass grafting; F, female; M, male; POAF, postoperative atrial fibrillation

**Table 2:** Pooled odds ratio or standardized mean difference of risk factors

Risk factors	No. of study	OR/SMD (95% CI)	Z value	*p* value	I^2^% (p)	Egger’s test, p
*Preoperative risk factors*						
Demographics						
Age (years)	8	1.10 (0.62–1.59)	4.46	< 0.001	0.0 (0.811)	0.658
Male	9	0.92 (0.79–1.08)	− 1.01	0.313	1.9 (0.419)	0.367
Female	9	1.08 (0.93–1.27)	1.01	0.313	1.9 (0.419)	0.367
BMI (kg/m^2^)	3	− 0.03 (− 0.10 to 0.04)	− 0.82	0.412	0.0 (0.956)	0.262
Clinical characteristics						
Mg^++^ (mg/dL)	2	− 0.37 (− 0.84 to 0.10)	− 1.55	0.121	74.9 (0.046)	NA
Ca^++^ (mg/dL)	2	− 0.91 (− 0.30 to 0.12)	− 0.86	0.398	0.0 (0.528)	NA
Serum Cr (mg/dL)	3	0.12 (0.05–0.19)	3.39	0.001	0.0 (0.826)	0.636
Hemoglobin (mg/dL)	3	− 0.10 (− 0.17 to − 0.03)	− 2.71	0.007	0.0 (0.846)	0.825
LVEF (%)	6	− 0.43 (− 0.43 to − 0.27)	− 3.43	0.001	71.4 (0.004)	0.553
Beta-blockers	8	0.89 (0.68–1.17)	− 0.83	0.405	35.9 (0.142)	0.260
ACEi	3	1.05 (0.91–1.21)	0.62	0.535	0.0 (0.927)	0.375
ACEi/ARB	2	0.43 (0.12–1.54)	− 1.30	0.195	65.4 (0.089)	NA
Nitrates	2	1.63 (0.86–3.02)	1.54	0.124	0.0 (0.528)	NA
Diuretics	2	1.33 (0.69–2.55)	0.86	0.390	0.0 (0.912)	NA
Statin	2	1.02 (0.57–1.82)	0.05	0.959	0.0 (0.367)	NA
CCB	4	1.22 (0.92–1.61)	1.37	0.171	19.8 (0.291)	0.310
Comorbidities						
MI	5	1.37 (0.96–1.96)	1.71	0.088	39.8 (0.156)	0.326
Essential hypertension	8	1.33 (1.62–1.52)	4.13	< 0.001	0.0 (0.581)	0.359
Diabetes	8	1.23 (0.96–1.56)	1.67	0.095	37.9 (0.127)	0.308
COPD	5	1.66 (1.13–2.43)	2.59	0.010	0.0 (0.494)	0.759
Renal failure	3	1.70 (1.14–2.55)	2.60	0.009	0.0 (0.928)	0.766
Dyslipidemia	4	1.47 (0.78–2.76)	1.20	0.232	82.2 (0.001)	0.225
Stroke	2	1.08 (0.58–2.00)	0.24	0.813	0.0 (0.708)	NA
CVD	2	1.60 (0.93–2.75)	1.68	0.628	0.0 (0.428)	NA
*Intraoperative risk factors*						
Number of grafts	5	− 0.09 (− 0.29 to 0.11)	− 0.86	0.393	49.3 (0.096)	0.500
Cross-clamp time (min)	4	0.06 (− 0.08 to 0.20)	0.82	0.415	1.1 (0.387)	0.946
Off-pump	2	1.37 (0.67–2.80)	0.87	0.386	56.1 (0.131)	NA
CPB time (min)	3	0.20 (0.04–0.36)	2.39	0.017	12.5 (0.319)	0.422
*Postoperative risk factors*						
IV inotrope	2	1.74 (1.50–2.02)	7.37	< 0.001	0.0 (0.551)	NA
Infection	3	2.45 (0.78–7.66)	1.54	0.123	68.38 (0.042)	0.877
Renal failure	3	3.94 (1.70–9.16)	3.19	0.001	0.0 (0.771)	0.582
Re-operation	2	6.41 (1.75–23.42)	2.81	0.005	0.0 (0.485)	NA
Mechanical ventilation (h)	2	0.26 (− 0.03 to 0.54)	1.78	0.076	0.0 (0.606)	NA

ACEi, angiotensin-converting enzyme inhibitor; ARB, angiotensin receptor blocker; BMI, body mass index; CCB, Ca^++^ channel blocker; CI, confidence interval; COPD, chronic obstructive pulmonary diseases; Cr, creatinine; CVD, cerebrovascular disease; IV, intravenous; LVEF, left ventricle ejection fraction; MI, myocardial infarction; NA, not available; OR, odds ratio; SMD, standardized mean difference

### Data analysis

The characteristics of the reviewed studies were summarized using descriptive statistics. The chi-square test was performed to confirm differences in POAF incidence according to CPB. For meta-analysis, the results of univariate analysis of individual risk factors were extracted by odds ratio (nominal variables such as sex and comorbidity) or mean with standard deviation (continuous variables such as age and hemoglobin) from nine reviewed articles. The effect size was calculated for each risk factor that had results reported in at least two articles to identify the trend of as many risk factors as possible.

The meta-analysis was conducted using Comprehensive Meta-Analysis software (version 3.0; Biostat, Englewood, NJ, USA). This was used to calculate pooled odds ratios and standardized mean differences with 95% confidence intervals. We utilized a random-effects model owing to the presumed heterogeneity between studies [31]. Heterogeneity in the results was judged by using the inverse variance index (I^2^) with its 95% confidence intervals and Q statistics (statistical significance at *p* < 0.05). If heterogeneity was high, possible cause were identified through a meta-ANOVA for the subgroup analysis [32]. Publication bias was calculated using Egger’s linear regression test [33].

## Results

### Description of studies

The characteristics of studies included in our analysis are summarized in Table 1. The studies were performed in eight different countries and were published between 1995 and 2018. In total, the studies included data on 4798 patients, and new-onset POAF after CABG was identified in 1555 (32.4%) of these patients.

The incidence rate of new-onset POAF ranged from 17.3 to 47.4% (Table 1). The median time to new-onset POAF was reported in four studies; it ranged between 2 and 4 days after CABG, with peak incidence on postoperative day 2. The mean age of patients without POAF after CABG (i.e., normal sinus rhythm after CABG) ranged from 61.2 to 66.0 years, while that of patients who developed new-onset POAF ranged from 63.9 to 71.0 years. The study population had a higher proportion of males (> 62.1%; Table 1). Based on the NOS, all studies included were of good quality.

### Preoperative risk factors for new-onset POAF after CABG

The following risk factors for new-onset POAF after CABG were identified in the meta-analysis (Table 2, Fig. 2, Additional file 1): old age (*p* < 0.001), high preoperative serum creatinine level (*p* = 0.001), low preoperative hemoglobin level (*p* = 0.007), a low left ventricular ejection fraction (LVEF; *p* = 0.001), essential hypertension (*p* < 0.001), COPD (*p* = 0.010), and renal failure (*p* = 0.009).

Subgroup analysis for high between-study heterogeneity was only required for the LVEF result (I^2^ = 71.4, *p* = 0.004; Table 3). The high heterogeneity was maintained on subgroup analysis. There were no differences in effect size according to publication year (Q = 0.58, degrees of freedom (*df*) = 1, *p* = 0.448) or sample size (Q = 1.65, *df* = 1, *p* = 0.199). However, there was a significant difference according to the country in which the study was performed (Q = 12.26, *df* = 1, *p* < 0.001), with low heterogeneity (I^2^ = 20.0, *p* = 0.289). On subgroup analysis, a low LVEF was a significant risk factor (*p* < 0.001) in Asian populations but not in European populations.

**Table 3:** Subgroup analysis of left ventricle ejection fraction according to study characteristics

Group	No. of study	SMD (95% CI)	Z-value	*p* value	I^2^% (p)	Q (p)
All studies	6	− 0.43 (− 0.67 to − 0.18)	− 3.43	0.001	71.4 (0.004)	
*Publication year*						
Before 2010	2	− 0.50 (− 0.80 to − 0.20)	− 3.28	0.001	0 (0.342)	0.58 (0.448)
After 2010	4	− 0.40 (− 0.72 to − 0.07)	− 2.38	0.017	81.2 (0.001)	
*No. of participants*						
≥ 200	2	− 0.36 (− 0.93 to 0.21)	− 1.24	0.217	91.8 (< 0.001)	1.65 (0.199)
< 200	4	− 0.48 (− 0.69 to − 0.27)	− 4.54	< 0.001	15.8 (0.313)	
*Study location*						
Europe	2	− 0.16 (− 0.42 to 0.10)	− 1.21	0.228	31.2 (0.228)	12.26 (< 0.001)
Asia	4	− 0.57 (− 0.76 to − 0.39)	− 6.04	< 0.001	20.0 (0.289)	

CI, confidence interval; SMD, standardized mean difference

### Intraoperative risk factors for new-onset POAF after CABG

The following intraoperative risk factors were reported: intra-aortic balloon pump use, type of graft used, and transfusion. Among these, only CPB (perfusion) time was retained as a significant risk factor on meta-analysis (*p* = 0.017; Table 2, Fig. 2).

To verify the specific effect of CPB time on new-onset POAF after CABG, we included only the outcomes from the seven studies (4445 patients) in which CPB use was clearly indicated [21–24, 26–28]. Among the 3533 patients in whom on-pump CABG was performed, new-onset POAF occurred in 1204 (34.1%) patients. By comparison, among the 912 patients in whom off-pump CABG was performed, new-onset POAF occurred in 262 (28.7%) patients. This difference in incidence rate of new-onset POAF was significant between the on-pump and off-pump CABG groups (χ^2^ = 9.39, *p* = 0.002).

### Postoperative risk factors for new-onset POAF after CABG

Among postoperative risk factors, inotrope use (*p* < 0.001), renal failure (*p* = 0.001), and re-operation (*p* = 0.005) were significantly associated with the incidence of new-onset POAF (Table 2, Fig. 2).

## Discussion

The findings of our meta-analysis are based on nine prospective studies regarding the onset of new-onset POAF after isolated CABG. The incidence rate of new-onset POAF ranged between 17.3 and 47.4%. Differences in the reported incidence rate across studies are likely related to differences in population characteristics, inclusion criteria, and diagnosis of AF. As an example, Tsai et al. [27] included patients who underwent isolated CABG, with and without CBP use, and identified POAF as a condition detected by electrocardiogram (EKG) telemetry monitoring or requiring anti-arrhythmic treatment. In contrast, Zangrillo et al. [23] included only patients who underwent isolated elective off-pump CABG and defined POAF as a condition detected by 12-lead EKG. Regardless of the variability among the included studies, the overall mean incidence of new-onset POAF of 32.4% across all participants is a matter of concern [8, 14] which points to the need to reduce the incidence of POAF by building consensus to minimize technical variations in the procedure, such as use of CPB and correcting risk factors for POAF such as preoperative anemia.

The time to new-onset of POAF ranged from 2 to 4 days after CABG. Although only four of the nine studies in our analysis reported on this outcome, the range agrees with previously published findings [8, 12]. This is an important perioperative period which includes the patient awakening from anesthesia, extubation, and postoperative care for the prevention of pulmonary complications. As patients are strictly monitored for EKG changes and vital signs during this period, this could be an optimal time for critical care staff to detect POAF and to provide appropriate management if POAF develops. The risk factors identified in our review can assist in the identification of patients at risk for new-onset POAF during this critical period after CABG. Based on our results, healthcare professionals should be aware that about 30% of patients will develop POAF within 4 days after CABG and that this risk is higher for older patients. They should also have knowledge of the other risk factors for new-onset of POAF after CABG, which are high preoperative serum creatinine and low hemoglobin level, low LVEF, essential hypertension, COPD, and preexisting renal failure, long CPB time, postoperative use of inotropes, postoperative renal failure, and re-operation. These risk factors identified in our review agree with previous results [4, 34, 35], presenting clear evidence of their importance. Based on these findings, it may be effective to design strategies for the timely screening of patients who are at high risk of new-onset POAF after CABG to provide patient-centered care according to their clinical trajectory.

As inflammation and cardiac ischemia are primary pathophysiological factors of POAF after CABG, CPB use during CABG may be an important factor to consider [12]. To overcome the limitation of relying solely on small-scale prospective cohort studies to evaluate the effect of CPB use, we performed additional analysis for CPB use. Although the use of CPB did not influence the occurrence of POAF in each reviewed article, the overall meta-analysis results did show that longer CPB time may be related to the development of new-onset POAF after CABG. The repercussions of excessive inflammation following CPB use are well known; however, the effect of CPB on mortality has not been conclusively resolved [16]. The impact of CPB use on the occurrence of POAF after CABG also needs further evaluation. A better understanding of CPB use for CABG would be specifically important as POAF is not only a transient complication of CABG but has long-term effects both in terms of mortality and risk of stroke [8–10]. Therefore, further research is essential to enable meta-analysis for variables including intra- and postoperative risk factors for new-onset of POAF after CABG.

Of note, our findings did not identify stroke as a significant risk factor for new-onset POAF, which is different from previous reports [34, 35]. In the studies included in our review, while postoperative neurologic conditions were presented in various forms including stroke, there was no specific explanation for each condition; as such, neurologic conditions could not be addressed and combined for meta-analysis. Other meaningful factors, such as peripheral vascular disease or the AF risk score, could not be analyzed quantitatively because of fragmentation of reported data or the use of different scales across studies [7, 13]. These reasons could explain differences in risk factors identified between our review and previous studies. Thus, there is a need for multicenter prospective studies that can identify the effects of the confirmed and controversial risk factors presented in our review and in prior studies.

Importantly, it is common for older patients who have a greater incidence of comorbidities, including renal failure and impaired left ventricle function, to undergo CABG [36, 37]. Presently, CABG is increasingly becoming a more widely accepted treatment option for older adults because of advances in anesthesia and surgical techniques and increased life expectancy [38]. Age is not a simple variable as it is also related to the development of health comorbidities and sex-specific differences in health. Hypertension, COPD, and renal dysfunction are representative chronic conditions in older adults [39]. Moreover, older females experience a dramatic increase in the risk of chronic diseases, including cardiovascular disease [40, 41]. Interestingly, based on the present results, older patients with essential hypertension, COPD, renal failure, a low hemoglobin level, and a low LVEF were vulnerable to new-onset POAF. In the current era of cardiac surgery, with more than half of the procedures being performed in patients ≥ 75 years of age [42, 43], proactive screening and better pre-, peri-, and postoperative management are necessary for older individuals undergoing CABG.

This study has several limitations which should be acknowledged in the interpretation of results. First, owing to the importance of new-onset POAF, articles that did not mention the presence or absence of preoperative AF were excluded. However, considering the types of AF, it would be more accurate to exclude only studies with patients who are being treated with anticoagulants. Second, data measured in intensive care units such as postoperative vital signs could not be systematically considered in our analysis. Although reported in some studies, these data could not be combined for meta-analysis owing to differences in reported variables and measurements used. Third, since meta-analysis for the continuous variables such as age and hemoglobin were performed using standardized mean difference in our study, it is not possible to provide a clear value to distinguish a risk group. Fourth, the possibility of publication bias cannot be completely excluded as the number of articles for each variable ranged between two and nine.

Despite these limitations, using findings based on prospective studies, we revealed that old age, a high preoperative serum creatinine level, a low LVEF in Asian populations, a low hemoglobin level, essential hypertension, COPD, renal failure, CPB use and duration of perfusion, use of postoperative inotropes, postoperative renal failure, and re-operation were significantly associated with new-onset POAF after CABG. These results may form the foundation for POAF surveillance efforts by healthcare providers. In addition, despite the existence of several studies devoted to risk model development, there is a clear need for further research to provide specific guidelines regarding risk factors for POAF. Therefore, it is still necessary to specify the characteristics of target patients (i.e., isolated CABG or on-pump CABG) and to comprehensively consider pre-, peri-, and postoperative risk factors.

## Conclusions

Careful stratification of patients to identify those in the high-risk group, using the criteria identified in this study, may lead to rapid recognition and treatment of new-onset POAF after CABG, reducing the risk of other complications and negative clinical outcomes. Our review highlights the high prevalence of new-onset POAF following CABG and the risk factors identified which could be included in a comprehensive screening tool. Our findings form a sound basis for guiding future multicenter prospective studies to strengthen the evidence base for risk of new-onset POAF after CABG. For healthcare professionals, strategies to monitor for and detect new-onset POAF should include management of preoperative risk factors, such as age-related health comorbidities, and a proactive management of peri- and postoperative complications. Larger multicenter cohort studies with greater power to detect associations between demographics, health factors, and new-onset POAF, as well as studies designed to address the limitations of previous research, may elucidate some of the yet unidentified risk factors of new-onset POAF after CABG.

## Supplementary Information

**Additional file 1.** The raw data of the significant continuous variables.

## Data Availability

All data generated or analyzed during this study are included in this published article.

## Abbreviations

AF: Atrial fibrillation
CABG: Coronary artery bypass grafting
CI: Confidence interval
COPD: Chronic obstructive pulmonary diseases
CPB: Cardiopulmonary bypass
EKG: Electrocardiogram
LVEF: Left ventricle ejection fraction
NOS: Newcastle–Ottawa Scale
POAF: Postoperative atrial fibrillation
