# Supplementary material for: Perioperative risk factors for new-onset postoperative atrial fibrillation after coronary artery bypass grafting: a systematic review
Source: BMC Cardiovasc Disord. 2021 Sep 3;21:418. doi: 10.1186/s12872-021-02224-x (PMC8414730; doi:10.1186/s12872-021-02224-x)
Supplement: Supplementary file 1 — Additional file 1. The raw data of the significant continuous variables. [file 12872_2021_2224_MOESM1_ESM.pdf]

Supplementary material. The raw data of the significant continuous variables

Reviewed articles	New-onset POAF (n)	Sinus rhythm (n)	Age (mean, SD)		preOP SCr[mg/dL] (mean, SD)		Hemoglobin (mean,SD)		preOP LVEF[%] (mean, SD)		CBP time[min] (mean, SD)	
			AF	SR	AF	SR	AF	SR	AF	SR	AF	SR
Mendes et al. (1995) [21]	57	111	[illegible]	[illegible]	[illegible]	[illegible]	[illegible]	[illegible]	[illegible]	[illegible]	[illegible]	[illegible]
Cerillo et al. (2003) [22]	33	74	70.2 ± 9.4	65.8 ± 9.8	[illegible]	[illegible]	[illegible]	[illegible]	50.5 ± 9.0	53.9 ± 9.7	94.4 ± 23.9	96.1 ± 29.5
Zangrillo et al. (2004) [23]	33	127	68.0 ± 8.8	64.0 ± 8.7	[illegible]	[illegible]	14 ± 1.7	14 ± 2.6	[illegible]	[illegible]	[illegible]	[illegible]
Akazawa et al. (2008) [24]	26	124	71.0 ± 8.0	66.0 ± 10.0	[illegible]	[illegible]	[illegible]	[illegible]	49.0 ± 14.0	57.0 ± 12.0	[illegible]	[illegible]
Wang et al. (2012) [25]	60	137	71.0 ± 11.0	64.0 ± 13.0	2.1 ± 2.1	1.8 ± 2.0	[illegible]	[illegible]	41.0 ± 11.0	48.0 ± 11.0	[illegible]	[illegible]
Koolen et al. (2013) [26]	1080	2068	69.8 ± 8.7	64.7 ± 9.8	1.12 ± 0.80	1.03 ± 0.68	8.6 ± 1.0	8.7 ± 1.0	[illegible]	[illegible]	[illegible]	[illegible]
Tsai et al. (2015) [27]	126	140	69.90 ± 11.62	61.84 ± 10.76	[illegible]	[illegible]	[illegible]	[illegible]	48.94 ± 15.19	58.19 ± 13.02	113.24 ± 58.88	98.86 ± 55.01
Vlahou et al. (2016) [28]	111	335	68.08 ± 8.5	63.05 ± 9.4	[illegible]	[illegible]	[illegible]	[illegible]	52.8 ± 12.6	53.6 ± 10.6	97.8 ± 36.1	91.1 ± 34.1
Daie et al. (2018) [29]	29	127	63.89 ± 8.91	61.19 ± 8.90	1.00 ± 0.36	1.00 ± 0.28	13.42 ± 1.35	13.68 ± 1.72	52.70 ± 10.34	54.73 ± 9.32	[illegible]	[illegible]

Abbreviations: POAF; postoperative atrial fibrillation, SD; standard deviation
